# New Frontiers in Anxiety Research: The Translational Potential of the Bed Nucleus of the Stria Terminalis

**DOI:** 10.3389/fpsyt.2019.00510

**Published:** 2019-07-17

**Authors:** Lindsay K. Knight, Brendan E. Depue

**Affiliations:** ^1^Interdisciplinary Program in Translational Neuroscience, University of Louisville, Louisville, KY, United States; ^2^Department of Psychological and Brain Sciences, University of Louisville, Louisville, KY, United States; ^3^Department of Anatomical Sciences and Neurobiology, University of Louisville, Louisville, KY, United States

**Keywords:** anxiety, BNST, bed nucleus of the stria terminalis, amygdala, anxiety disoders, fMRI

## Abstract

After decades of being overshadowed by the amygdala, new perspectives suggest that a tiny basal forebrain region known as the bed nucleus of the stria terminalis (BNST) may hold key insights into understanding and treating anxiety disorders. Converging research indicates that the amygdala and BNST play complementary but distinct functional roles during threat processing, with the BNST specializing in the detection of a potential threat to maintain hypervigilance and anxiety, while the amygdala responds to the perceived presence of an aversive stimulus (i.e., fear). Therefore, given that human anxiety is largely driven by future-oriented hypothetical threats that may never occur, studies involving the BNST stand at the forefront of essential future research with the potential to bring about profound insights for understanding and treating anxiety disorders. In this article, we present a narrative review on the BNST, summarizing its roles in anxiety and the stress response and highlighting the most recent advances in the clinical realm. Furthermore, we discuss oversights in the current state of anxiety research and identify avenues for future exploration.

## Introduction

Anxiety disorders are currently the most prevalent subgroup of mental disorders in most Western societies, with nearly one in three lifetime incidence in the USA (1, 2). These disorders are not only pervasive, but are frequently chronic and a leading cause of disability worldwide (3). While significant progress has been made in understanding the neural circuitry of threat processing in preclinical studies, these mechanistic advances have not translated to widely efficacious therapies. Promising new treatments either have turned out to be only moderately effective or have induced adverse side effects, limiting applicability in clinical practice (3–5).

To date, anxiety disorder research has primarily fixated on the amygdala, with nearly 5,000 human neuroimaging studies alone detailing its central role in emotion processing and threat detection (6). This line of work has led to well-supported conclusions that anxiety disorders can, in part, be attributed to hyper-responsivity of the amygdala to perceived threat (7), as well as dysregulated prefrontal control over amygdala reactivity due to altered structural or functional connectivity (8). Yet discouragingly, this same ventromedial prefrontal (vmPFC) to amygdala circuit dysfunction has also been proposed as a model for many other disorders ranging from depression (9) to psychopathy (10). While many psychiatric and mood disorders undoubtedly share some semblance of dysregulated emotion processing, explaining this common finding, it is unlikely that this single pathway represents such a broad etiology that could account for the heterogeneous symptomatology and phenotypic dysfunction seen across disorders or even within a single disorder. Although revolutionary in its initial discovery, this explanation of anxiety disorders now stands as an oversimplification that is ultimately hindering our understanding. The field is in need of the next iteration of specificity. Fortuitously, emerging research suggests that a tiny and lesser-known basal forebrain region may bring about a new wave of insights and opportunities for the development of novel therapeutics. Enter: the bed nucleus of the stria terminalis (BNST).

## Distinguishing Anxiety From Fear

Anxiety can be defined as a prolonged state of apprehension brought on by an uncertain or unpredictable prospective threat. In rodents, anxiety-like behaviors can be elicited by physically distant threats such as a predator in the environment, or diffuse contextual threats like a brightly lit open space. While comparable situations can indeed be anxiety provoking for humans (e.g., dark enclosed spaces), in general, humans are much more prone to encounter *psychological* stressors. Thus, an anxious emotional state can be triggered by ambiguously threatening stimuli or even by internally generated thoughts of real or imagined prospective threats. While the term “anxiety” is often colloquially used interchangeably with “fear,” more precisely, fear describes a phasic response to the presence of an immediate and identifiable threat (6). However, it should be noted that perception is critical, as a threatening stimulus that is perceived as present or even imagined can activate a fear response.

Corresponding to this psychological dissociation between fear and anxiety, converging evidence suggests that two partially segregated neural circuits support these divergent responses (11, 12). Spearheaded by Davis and Walker, a highly influential model theorizes that the amygdala underlies phasic responses to explicit threats, supporting feelings of fear, while the BNST, considered part of the “extended amygdala,” is thought to mediate more sustained responses to unpredictable, ambiguous, or diffuse threats, thus underlying persistent states of anticipation or hypervigilance and promoting feelings of anxiety (11). In further support of these distinct functional roles, studies in rodents show that lesioning the amygdala eliminates conditioned fear to auditory (13) and visual conditioned stimuli (14) and reduces fear-potentiated startle (15), but does not alter anxiety-like behavior in an elevated plus maze (15) or anxiety-like responses to bright light or corticotropin-releasing hormone (CRH) injection (14). Conversely, lesioning the BNST attenuates anxiety-like responses (16–20) and alters cortisol release (21) but, importantly, does not affect conditioned fear (14, 17, 19, 20).

While there is a general consensus for the involvement of the BNST in anxiety processing, the mechanisms are less well-understood due to the complexity of the BNST structure and the wide variety of the neurotransmitters it expresses, including GABA, glutamate, noradrenaline (NA), serotonin (5-HT), and CRH, among others (22). The literature suggests that glutamatergic and GABAergic neuronal populations have opposing influences, with glutamate promoting anxiogenic effects and GABA inducing a reduction in anxiety (23). Although the GABAergic population dominates in the BNST (24), in many cases, the glutamatergic subpopulation exerts a greater overall influence, in part due to higher intrinsic excitability and altered responsivity to NA (23). The interaction between NA and 5-HT is also believed to contribute to anxiety, with the majority of evidence suggesting that anxiety disorders are characterized by underactivation of serotonergic function and overactivation or complex dysregulation of noradrenergic function (25). In adaptive anxiety, release of CRH is met by inhibition *via* 5-HT, which aids in decreasing reactivity of the BNST and regulating the stress response. Furthermore, while NA ramps up autonomic arousal, raising heart rate, and increasing memories of aversive contexts, 5-HT acts to decrease such memories. Thus, dysregulation of this mutually inhibitory system can lead to increased vigilance and aversive behavior due to overactive NA (26) and decreased inhibition of stress reactivity due to a hyporesponsive 5-HT system (25).

CRH has repeatedly been identified as an important contributor to fear and anxiety behavior and is largely expressed in stress-related brain regions, including the amygdala and BNST. Once more, this points to the BNST as not only a mediator of anxious feelings and behaviors but also a central modulator of the stress response (27). The BNST is ideally situated in the brain to stimulate allostatic changes through its dense connections with the paraventricular nucleus (PVN) of the hypothalamus, the primary node of the hypothalamic-pituitary–adrenal (HPA) axis that initiates the stress response and regulates cortisol release. Perhaps even more compelling, evidence suggests that the BNST’s position is important for coordinating neuroendocrine and behavioral responses (28, 29). Very few limbic forebrain regions provide direct innervation to the PVN, but the BNST appears to serve as a point of convergence between these higher-order regions and HPA effector neurons. Furthermore, rather than merely relaying these signals, the BNST has been shown to dynamically integrate information from multiple upstream sources, including the medial prefrontal cortex and hippocampus, and to modulate the downstream neuroendocrine and behavioral responses during stress (28, 29). Thus, differences in the structural or functional connectivity of the prefrontal–BNST or hippocampal–BNST pathways could bias an individual towards different coping styles or alter susceptibility toward anxiety and other stress-related disorders. With this understanding of the BNST’s role in mediating anxiety and the stress response, a renewed emphasis has been placed on the investigation of the human BNST throughout the past decade, although research in humans, and specifically in relation to anxiety and other stress-related disorders, is still in its infancy.

## The Human BNST

**Figure 1:**
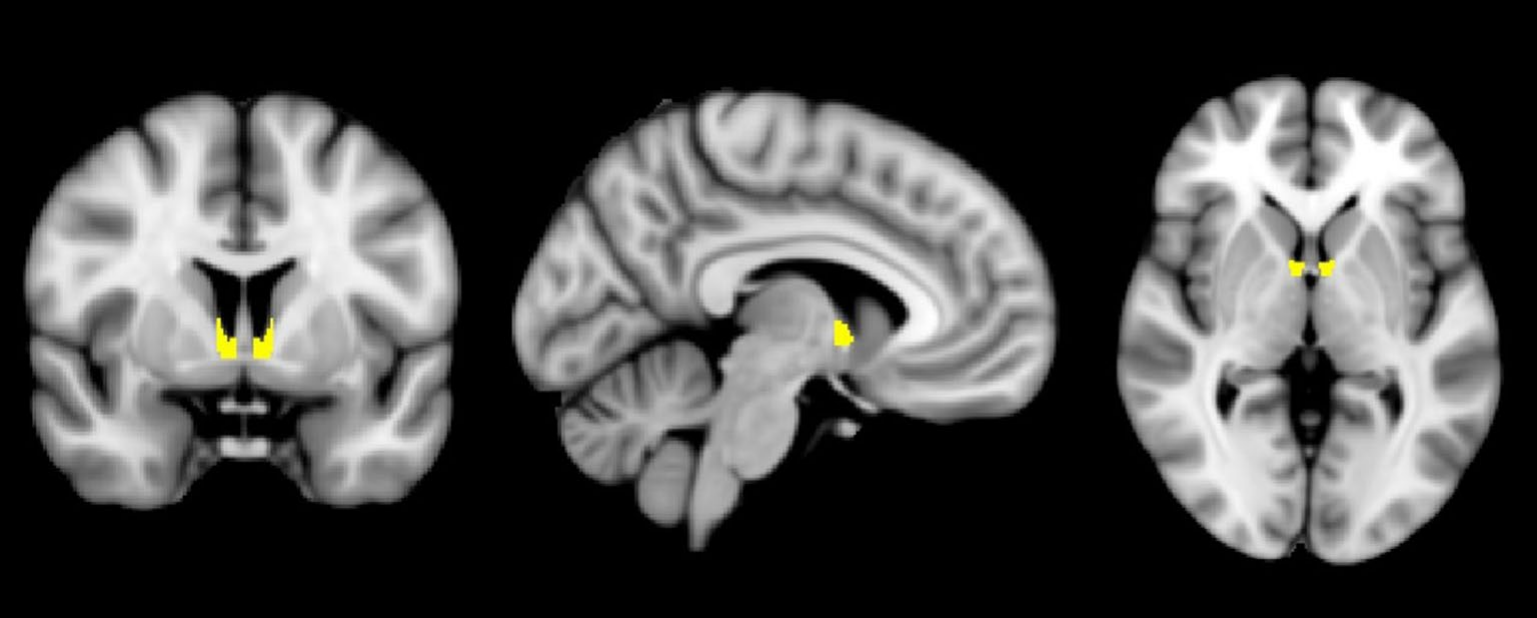
The human bed nucleus of the stria terminalis (BNST). A mask of the BNST [from Ref. (6) ] is shown highlighted in yellow, overlaid on a standardized average brain.

Less is known about the human BNST (**Figure 1**), in part due to the combination of its small structure size and the relatively low spatial resolution of standard functional MRI (fMRI). With just 12–18 subnuclei comprising the BNST (27) and at approximately 190 mm^3^—the size of a sunflower seed—the BNST is so small that many human neuroimaging studies have qualified their reported results with statements such as “a region overlapping” or “consistent with” the BNST (6). However, with recent advances in neuroimaging technology, including improvements that permit a 27× increase in spatial resolution (e.g., 3 to 1 mm^3^), new opportunities await to reinvigorate the investigation about the distinction between fear and anxiety in humans and the relative importance and influence of the BNST in cognitive health and dysfunction.

Studies that have begun to approach these questions in humans have described complementary findings to the pioneering work of Davis and Walker. For example, work by Alvarez and colleagues reported a similar dissociation in the functional roles of the amygdala and BNST using a combination of cued and contextual threats. During fMRI scanning, participants were placed in three prerecorded virtual reality environments: a restaurant, casino, or bank. One environment served as a predictable threat context in which electric shocks were consistently delivered following an auditory tone. In the other two contexts, the tone was meaningless, with shocks being administered in an unsignaled or semi-random manner in one environment (unpredictable threat) and no shocks being delivered in the control context. Results showed that amygdala activity transiently increased at the onset of both threat contexts, but only the unpredictable threat context yielded sustained activity in the BNST (30), supporting previous animal models of phasic and sustained fear. Additional investigations in humans have helped uncover a more nuanced role for the BNST, suggesting that rather than simply mediating sustained responses to threats, the BNST appears to exhibit a specialized role in detecting potential threats when the specifics of the threat are uncertain. In another study, participants viewed videos of a line fluctuating in height over time and were told that each time the line exceeded a certain threshold, they would accumulate an electric shock to be delivered after the task (but in fact, were never actually shocked). During this time of anxiously anticipating future shocks, the authors reported robust BNST activity, but the amygdala was said to show minimal task-modulated activity even at exploratory statistical thresholds (31). As a result, the BNST was given a new title of “threat monitoring,” and in support of this notion, further reports demonstrated the BNST’s ability to track threat proximity in both the physical sense (e.g., monitoring the distance of an approaching tarantula) (32) and the psychological sense (e.g., tracking the likelihood of threat occurrence) (31).

Later, studies subsequently sought to separate out the responses related to the anticipation or monitoring of a prospective threat, relative to actual threat confrontation (i.e., presentation of aversive stimulus). In two closely related but independent studies, BNST activity was found to be significantly elevated during uncertain threat anticipation, while it was the amygdala that exhibited a significant response during the aversive outcome (12, 33). In sum, these findings suggest that a regional dissociation can be attributed to the BNST playing a role in helping to detect a potential threat and maintain hypervigilance until threat encounter or situational resolve, while the amygdala preferentially responds to the actual presence of an aversive stimulus, mediating instantaneous responses during acute danger. Therefore, given that human anxiety is largely driven by future-oriented hypothetical threats that may never occur, studies involving the BNST stand at the forefront of essential future research.

## Oversights and Opportunities

Although this relationship between uncertainty about future adverse events and anxiety makes intuitive sense, this conceptualization of anxiety has not been reflected in many neuroimaging investigations aimed at elucidating the neurocircuitry of clinical anxiety disorders. This is principally true in studies investigating how emotion is regulated. Dysregulated emotion is a hallmark among many psychiatric disorders including anxiety disorders, and consequently, a strong focus has been placed on uncovering the neural mechanisms supporting effective emotion regulation (ER) due its significance and potential applicability transdiagnostically. Typically, ER is studied in the context of individuals attempting to volitionally control their emotional response to explicitly cued and overtly displayed pictorial stimuli (negative scenes or faces), through reappraisal or distancing/suppression strategies (34–36). This work indicates that the degree of regulating subjective negative emotion is dependent upon the strength of functional and structural connections between the vmPFC and the amygdala, which is likely mediated by higher-order lateral prefrontal regions to ultimately downregulate amygdala activity through top–down goal-directed behavior.

However, three critical barriers arise when this line of research is intended to specifically elucidate ER mechanisms in the context of anxiety disorders. First, many ER studies utilize stimuli meant to induce disgust or general negative affect rather than simulate ecologically relevant threats. Second, because the predominant focus of ER research has been centered on emotion control during the *overt* display of such aversive stimuli, these tasks are not capturing the psychological processes at the heart of anxious pathology—namely, the anticipatory cognitive and affective processes in the face of uncertain or unpredictable threats—and are instead essentially uncovering mechanisms needed to regulate general negative affect or disgust *after* a concrete stimulus has been presented. In light of this, recent studies are attempting to model threat anticipation more precisely to explicate the complex underlying neural circuitry (37). Furthermore, other lines of research are deriving more nuanced views in how attentional control may modulate anxiety-potentiated coupling between medial prefrontal and amygdala circuitry (38). Nevertheless, the field remains in critical need of work that definitively targets *anxiety* regulation. Finally, despite growing research demonstrating the BNST as a primary mediator of both anticipatory anxiety and the stress response, the BNST is essentially absent from ER literature. As a result, many crucial outstanding questions remain: How does the brain regulate thought and feeling in anticipation of uncertain and unpredictable threats? If the amygdala can be downregulated after a stimulus has been presented, can the BNST also be downregulated before stimulus presentation? If so, what are the mechanisms and does this downregulation reduce subjective feelings of anxiety? Does this then subsequently change processing of the overt stimulus?

## Clinical Implications

Anxiety disorders are characterized by both excessive fear and anxiety. However, elucidating the mechanisms of sustained anxious states and regulation of the stress response, both processes mediated by the BNST, appear to be especially relevant and not just in the case of generalized anxiety disorder (GAD). For example, individuals with posttraumatic stress disorder (PTSD) not only suffer from conditioned fear to cues that evoke traumatic memories, but they also exhibit persistent symptoms of sustained anxiety (e.g., hypervigilance). Similarly, in panic disorder (PD), although a hallmark is the experience of panic attacks, another key element is anxiety caused by persistent apprehension and continuous worry about the recurrence of future panic attacks (39). Even specific phobia, the prototypical “fear disorder,” involves episodes of sustained anxiety when anticipating a future confrontation with their phobic fear (39). Finally, intolerance of uncertainty, or an inability to cope with potential negative outcomes, is not only an established hallmark of GAD but may also be a transdiagnostic feature of obsessive–compulsive disorder (OCD), such that compulsions and ritualistic behaviors are performed as a means to reduce this distress (40).

Evidence from human neuroimaging studies reinforces the role of the BNST across anxiety disorder subtype. One study in GAD patients found higher arousal and increased activation in the BNST when exposed to a gambling game with high monetary uncertainty (41). Similarly, relative to healthy controls, GAD patients exhibited enhanced phasic activity in the amygdala and heightened sustained activity in the BNST when faced with a temporally unpredictable threat exposure involving human screams (42). Utilizing the same experimental paradigm, Brinkmann et al. (43, 44) found corresponding results in both PTSD and PD, with patients displaying sustained activation in the BNST during unpredictable anticipation of aversive sounds, relative to controls. Human neuroimaging investigations have additionally explored the role of the BNST in patients with specific phobia when anxiously anticipating the presentation of phobogenic stimuli (e.g., spiders). Under conditions of unpredictable sustained anticipation, patients showed increased activation in anterior cingulate cortex and once more, the BNST (45, 46), while the predictable phasic fear condition was associated with elevated amygdala activity (46). Together, these studies further strengthen the case for distinct functionality of the amygdala and BNST and indicate that heightened and prolonged reactivity of the BNST may be a contributing factor to clinical anxiety disorders.

Although very preliminary, small clinical case studies suggest that being able to selectively regulate BNST activity could have profound effects on anxious propensities and predispositions. In a double-blind, randomized crossover trial, deep brain stimulation (DBS) in the BNST showed to be effective in reducing obsessions and compulsions in OCD patients. In this report, it was noted that beneficial effects on mood and anxiety were observed first, before apparent changes in obsessions or compulsions, suggesting that these initial anxiolytic effects may subsequently drive the attenuation of OCD symptoms (47). Similarly, BNST DBS was used in a patient who had battled remitting and relapsing anorexia nervosa since adolescence (over 40 years in total), as well as concurrent major depressive disorder (MDD). Following bilateral BNST implantation, improvement was gradual but incredibly profound. Nine months after surgery, the patient was released from the psychiatric ward after nearly a 4-year stay, and tube feeding for her eating disorder was discontinued. The patient reported that all of her anxiety concerning food and eating had essentially vanished, and her food intake had become more stable. In the patient’s own words, despite the absence of anxious or obsessive thoughts, she continued to eat just enough to keep her weight stable out of habit, although she was now motivated to begin behavioral training to break this pattern (48).

Continued research on the BNST may additionally uncover insights into the onset and prevalence in anxiety disorders. Anatomically, the BNST is a sexually dimorphic structure, and although this adds an additional layer of complexity to research, these structural differences may help explain the gender disparity that exists in the prevalence of anxiety disorders and other stress-related psychiatric disorders. Interestingly, however, the BNST does not show strong sexual differentiation at birth, but rather appears to develop sexual dimorphism around puberty (49). This late divergence in BNST volume between men and women may be a general characteristic of the BNST and, if so, curiously coincides with the earliest onset of many anxiety disorders. Together, these observations offer yet a few more motivations for continued investigation of the BNST structure and function in humans.

## Conclusions

Progress in understanding the pathogenesis of anxiety and in identifying neural signatures that differentiate affected versus nonaffected individuals is critically dependent upon our ability to develop relevant models of anxiety. The crux of anxiety concerns uncertain and unpredictable threats, and therefore, the first essential step is to develop lab paradigms that psychologically elicit anxiety in an ecologically valid manner. At the same time, while the segregation between fear and anxiety is important in our theoretical approach to parse out the specific roles of regions such as the BNST, it is hard to image a real-life threatening scenario that solely depends on the actions of a single structure. Thus, in our continued effort to uncover the relative importance and influence of the BNST, we must also continue to explore the intricacies in which regions dynamically communicate within larger circuits and networks. Higher-order cognition undoubtedly requires cooperative activity from disparate regions and integration between distributed brain networks (50). Moreover, network organization is known to be temporally dynamic, whereby some regions may flexibly shift their functional connectivity to affiliate more strongly with some networks than others depending on the emotional state and current task demands (51, 52). For example, the amygdala is known to interact with the salience and cingulo-opercular networks in states of anxiety (53, 54). Investigations of such relationships with the BNST are rare (but see for example 51); however, it stands to reason that if the BNST represents a more tonic state in threat processing, this would likely be reflected in a strengthened relationship with higher-order attentional states (55). The approach of cognitive network neuroscience, therefore, aims to reconcile the seemingly opposing perspectives of functional segregation and functional integration, by investigating how networks, and regions within networks, dynamically communicate to support optimal processing (56). In sum, many avenues of research suggest that we are well on our way to untangling these intricacies, and we can be optimistic that the next decade of research will bring great strides in anxiety research and the neural bases of psychopathology, in part thanks to the untapped potential of the BNST.

## Author Contributions

LK wrote the manuscript. BD contributed to the conception of the manuscript, critically discussed its content and provided revisions.

## Funding

This work was supported by a Brain and Behavior Research Foundation (NARSAD) YI Grant [#OGMB180914] awarded to BD.

## Conflict of Interest Statement

The authors declare that the research was conducted in the absence of any commercial or financial relationships that could be construed as a potential conflict of interest.

## References

[1] Kessler RC Petukhova M Sampson NA Zaslavsky AM Wittchen HU Twelve-month and lifetime prevalence and lifetime morbid risk of anxiety and mood disorders in the United States. Int J Methods Psychiatr Res (2012) 21(3):169–84. 10.1002/mpr.1359 PMC4005415 22865617

[2] Craske MG Stein MB Eley TC Milad MR Holmes A Rapee RM Anxiety disorders. Nat Rev Dis Primers (2017) 3:17100. 10.1038/nrdp.2017.24 29239346

[3] Griebel G Holmes A 50 years of hurdles and hope in anxiolytic drug discovery. Nat Rev Drug Discov (2013) 12(9):667–87. 10.1038/nrd4075 PMC4176700 23989795

[4] Hyman SE Psychiatric drug development: diagnosing a crisis. In: Cerebrum: the Dana forum on brain science. New York, NY: Dana Foundation (2013). PMC3662213 23720708

[5] LeDoux JE Pine DS Using neuroscience to help understand fear and anxiety: a two-system framework. Am J Psychiatry (2016) 173(11):1083–93. 10.1176/appi.ajp.2016.16030353 27609244

[6] Avery SN Clauss JA Blackford JU The human BNST: functional role in anxiety and addiction. Neuropsychopharmacology (2016) 41(1):126–41. 10.1038/npp.2015.185 PMC4677124 26105138

[7] Etkin A Wager TD Functional neuroimaging of anxiety: a meta-analysis of emotional processing in PTSD, social anxiety disorder, and specific phobia. Am J Psychiatry (2007) 164(10):1476–88. 10.1176/appi.ajp.2007.07030504 PMC3318959 17898336

[8] Quirk GJ Beer JS Prefrontal involvement in the regulation of emotion: convergence of rat and human studies. Curr Opin Neurobiol (2006) 16(6):723–7. 10.1016/j.conb.2006.07.004 17084617

[9] Johnstone T van Reekum CM Urry HL Kalin NH Davidson RJ Failure to regulate: counterproductive recruitment of top–down prefrontal–subcortical circuitry in major depression. J Neurosci (2007) 27(33):8877–84. 10.1523/JNEUROSCI.2063-07.2007 PMC6672169 17699669

[10] Blair RJR The amygdala and ventromedial prefrontal cortex in morality and psychopathy. Trends Cogn Sci (2007) 11(9):387–92. 10.1016/j.tics.2007.07.003 17707682

[11] Davis M Walker DL Miles L Grillon C Phasic vs sustained fear in rats and humans: role of the extended amygdala in fear vs anxiety. Neuropsychopharmacology (2010) 35(1):105–35. 10.1038/npp.2009.109 PMC2795099 19693004

[12] Naaz F Knight LK Depue BE Explicit and ambiguous threat processing: functionally dissociable roles of the amygdala and bed nucleus of the stria terminalis. J Cogn Neurosci (2019) 31:543–59. 10.1162/jocn_a_01369 30605004

[13] Zimmerman JM Rabinak CA McLachlan IG Maren S The central nucleus of the amygdala is essential for acquiring and expressing conditional fear after overtraining. Learn Mem (2007) 14(9):634–44. 10.1101/lm.607207 PMC1994080 17848503

[14] Walker DL Davis M Double dissociation between the involvement of the bed nucleus of the stria terminalis and the central nucleus of the amygdala in startle increases produced by conditioned versus unconditioned fear. J Neurosci (1997) 17(23):9375–83. 10.1523/JNEUROSCI.17-23-09375.1997 PMC6573581 9364083

[15] Ventura-Silva AP Melo A Ferreira AC Carvalho MM Campos FL Sousa N Excitotoxic lesions in the central nucleus of the amygdala attenuate stress-induced anxiety behavior. Front Behav Neurosci (2013) 7:32. 10.3389/fnbeh.2013.00032 23626528 PMC3630370

[16] Fendt M Endres T Apfelbach R Temporary inactivation of the bed nucleus of the stria terminalis but not of the amygdala blocks freezing induced by trimethylthiazoline, a component of fox feces. J Neurosci (2003) 23(1):23–8. 10.1523/JNEUROSCI.23-01-00023.2003 PMC6742150 12514197

[17] Goode TD Ressler RL Acca GM Miles OW Maren S Bed nucleus of the stria terminalis regulates fear to unpredictable threat signals. eLife (2019) 8:e46525. 10.7554/eLife.46525 30946011 PMC6456295

[18] Hammack SE Richey KJ Watkins LR Maier SF Chemical lesion of the bed nucleus of the stria terminalis blocks the behavioral consequences of uncontrollable stress. Behav Neurosci (2004) 118(2):443. 10.1037/0735-7044.118.2.443 15113272

[19] Waddell J Morris RW Bouton ME Effects of bed nucleus of the stria terminalis lesions on conditioned anxiety: aversive conditioning with long-duration conditional stimuli and reinstatement of extinguished fear. Behav Neurosci (2006) 120(2):324. 10.1037/0735-7044.120.2.324 16719697

[20] Zimmerman JM Maren S The bed nucleus of the stria terminalis is required for the expression of contextual but not auditory freezing in rats with basolateral amygdala lesions. Neurobiol Learn Mem (2011) 95(2):199–205. 10.1016/j.nlm.2010.11.002 21073972 PMC3050017

[21] Sullivan GM Apergis J Bush DEA Johnson LR Hou M Ledoux JE Lesions in the bed nucleus of the stria terminalis disrupt corticosterone and freezing responses elicited by a contextual but not by a specific cue-conditioned fear stimulus. Neuroscience (2004) 128:7–14. 10.1016/j.neuroscience.2004.06.015 15450349

[22] Forray MI Gysling K Role of noradrenergic projections to the bed nucleus of the stria terminalis in the regulation of the hypothalamic–pituitary–adrenal axis. Brain Res Rev (2004) 47(1–3):145–60. 10.1016/j.brainresrev.2004.07.011 15572169

[23] Gungor NZ Yamamoto R Pare D Glutamatergic and gabaergic ventral BNST neurons differ in their physiological properties and responsiveness to noradrenaline. Neuropsychopharmacology (2018) 43:2126–33. 10.1038/s41386-018-0070-4 PMC6098041 29704000

[24] Kash TL Pleil KE Marcinkiewcz CA Lowery-Gionta EG Crowley N Mazzone C Neuropeptide regulation of signaling and behavior in the BNST. Mol Cells (2015) 38(1):1. 10.14348/molcells.2015.2261 25475545 PMC4314126

[25] Ressler KJ Nemeroff CB Role of serotonergic and noradrenergic systems in the pathophysiology of depression and anxiety disorders. Depress Anxiety (2000) 12(S1):2–19. 10.1002/1520-6394(2000)12:1+<2::AID-DA2>3.3.CO;2-W 11098410

[26] Ashwani A Tarun K Ajay M Anil H Anxiety disorders: a review. IRJP (2011) 2:18–23.

[27] Lebow MA Chen A Overshadowed by the amygdala: the bed nucleus of the stria terminalis emerges as key to psychiatric disorders. Mol Psychiatry (2016) 21(4):450. 10.1038/mp.2016.1 26878891 PMC4804181

[28] Radley JJ Sawchenko PE A common substrate for prefrontal and hippocampal inhibition of the neuroendocrine stress response. J Neurosci (2011) 31(26):9683–95. 10.1523/JNEUROSCI.6040-10.2011 PMC3197245 21715634

[29] Radley JJ Johnson SB Anteroventral bed nuclei of the stria terminalis neurocircuitry: towards an integration of HPA axis modulation with coping behaviors. Psychoneuroendocrinology (2017) 31(26):9683–95. 10.1016/j.psyneuen.2017.12.005 PMC5878723 29395488

[30] Alvarez RP Chen G Bodurka J Kaplan R Grillon C Phasic and sustained fear in humans elicits distinct patterns of brain activity. Neuroimage (2011) 55(1):389–400. 10.1016/j.neuroimage.2010.11.057 21111828 PMC3100535

[31] Somerville LH Whalen PJ Kelley WM Human bed nucleus of the stria terminalis indexes hypervigilant threat monitoring. Biol Psychiatry (2010) 68(5):416–24. 10.1016/j.biopsych.2010.04.002 PMC2921460 20497902

[32] Mobbs D Yu R Rowe JB Eich H Feldman Hall O Dalgleish T Neural activity associated with monitoring the oscillating threat value of a tarantula. Proc Natl Acad Sci (2010) 107(47):20582–6. 10.1073/pnas.1009076107 PMC2996708 21059963

[33] Klumpers F Kroes MC Baas J Fernández G How human amygdala and bed nucleus of the stria terminalis may drive distinct defensive responses. J Neurosci (2017) 37(40):9465–56. 10.1523/JNEUROSCI.3830-16.2017 PMC6596617 28893930

[34] Ochsner KN Bunge SA Gross JJ Gabrieli JD Rethinking feelings: an FMRI study of the cognitive regulation of emotion. J Cogn Neurosci (2002) 14(8):1215–29. 10.1162/089892902760807212 12495527

[35] Ochsner KN Ray RD Cooper JC Robertson ER Chopra S Gabrieli JD For better or for worse: neural systems supporting the cognitive down-and up-regulation of negative emotion. Neuroimage (2004) 23(2):483–99. 10.1016/j.neuroimage.2004.06.030 15488398

[36] Depue BE Orr JM Smolker HR Naaz F Banich MT The organization of right prefrontal networks reveals common mechanisms of inhibitory regulation across cognitive, emotional, and motor processes. Cereb Cortex (2015) 26(4):1634–46. 10.1093/cercor/bhu324 PMC4785949 25601236

[37] Grupe DW Nitschke JB Uncertainty and anticipation in anxiety: an integrated neurobiological and psychological perspective. Nat Rev Neurosci (2013) 14(7):488. 10.1038/nrn3524 23783199 PMC4276319

[38] Robinson OJ Krimsky M Lieberman L Vytal K Ernst M Grillon C Anxiety-potentiated amygdala–medial frontal coupling and attentional control. Transl Psychiatry (2016) 6(6):e833. 10.1038/tp.2016.105 27271859 PMC4931603

[39] Grillon C Models and mechanisms of anxiety: evidence from startle studies. Psychopharmacology (2008) 199(3):421–37. 10.1007/s00213-007-1019-1 PMC2711770 18058089

[40] Holaway RM Heimberg RG Coles ME A comparison of intolerance of uncertainty in analogue obsessive–compulsive disorder and generalized anxiety disorder. J Anxiety Disord (2006) 20(2):158–74. 10.1016/j.janxdis.2005.01.002 16464702

[41] Yassa MA Hazlett RL Stark CE Hoehn-Saric R Functional MRI of the amygdala and bed nucleus of the stria terminalis during conditions of uncertainty in generalized anxiety disorder. J Psychiatr Res (2012) 46(8):1045–52. 10.1016/j.jpsychires.2012.04.013 PMC3893050 22575329

[42] Buff C Brinkmann L Bruchmann M Becker MP Tupak S Herrmann MJ Activity alterations in the bed nucleus of the stria terminalis and amygdala during threat anticipation in generalized anxiety disorder. Soc Cogn Affect Neurosci (2017) 12(11):1766–74. 10.1093/scan/nsx103 PMC5714227 28981839

[43] Brinkmann L Buff C Feldker K Tupak SV Becker MPI Herrmann MJ Distinct phasic and sustained brain responses and connectivity of amygdala and bed nucleus of the stria terminalis during threat anticipation in panic disorder. Psychol Med (2017a) 47(15):2675–88. 10.1017/S0033291717001192 28485259

[44] Brinkmann L Buff C Neumeister P Tupak SV Becker MP Herrmann MJ Dissociation between amygdala and bed nucleus of the stria terminalis during threat anticipation in female post-traumatic stress disorder patients. Hum Brain Mapp (2017b) 38(4):2190–205. 10.1002/hbm.23513 PMC6866898 28070973

[45] Straube T Mentzel HJ Miltner WH Waiting for spiders: brain activation during anticipatory anxiety in spider phobics. Neuroimage (2007) 37(4):1427–36. 10.1016/j.neuroimage.2007.06.023 17681799

[46] Münsterkötter AL Notzon S Redlich R Grotegerd D Dohm K Arolt V Spider or no spider? Neural correlates of sustained and phasic fear in spider phobia. Depress Anxiety (2015) 32(9):656–63. 10.1002/da.22382 26115440

[47] Luyten L Hendrickx S Raymaekers S Gabriëls L Nuttin B Electrical stimulation in the bed nucleus of the stria terminalis alleviates severe obsessive–compulsive disorder. Mol Psychiatry (2016) 21(9):1272. 10.1038/mp.2015.124 26303665

[48] Blomstedt P Naesström M Bodlund O Deep brain stimulation in the bed nucleus of the stria terminalis and medial forebrain bundle in a patient with major depressive disorder and anorexia nervosa. Clin Case Rep (2017) 5(5):679–84. 10.1002/ccr3.856 PMC5412827 28469875

[49] Chung WC De Vries GJ Swaab DF Sexual differentiation of the bed nucleus of the stria terminalis in humans may extend into adulthood. J Neurosci (2002) 22(3):1027–33. 10.1523/JNEUROSCI.22-03-01027.2002 PMC6758506 11826131

[50] Medaglia JD Lynall ME Bassett DS Cognitive network neuroscience. J Cogn Neurosci (2015) 27(8):1471–91. 10.1162/jocn_a_00810 PMC4854276 25803596

[51] McMenamin BW Langeslag SJ Sirbu M Padmala S Pessoa L Network organization unfolds over time during periods of anxious anticipation. J Neurosci (2014) 34(34):11261–73. 10.1523/JNEUROSCI.1579-14.2014 PMC4138337 25143607

[52] Pessoa L Understanding emotion with brain networks. Curr Opin Behav Sci (2018) 19:19–25. 10.1016/j.cobeha.2017.09.005 29915794 PMC6003711

[53] Etkin A Prater KE Schatzberg AF Menon V Greicius MD Disrupted amygdalar subregion functional connectivity and evidence of a compensatory network in generalized anxiety disorder. Arch Gen Psychiatry (2009) 66(12):1361–72. 10.1001/archgenpsychiatry.2009.104 PMC12553334 19996041

[54] Sylvester CM Corbetta M Raichle ME Rodebaugh TL Schlaggar BL Sheline YI Functional network dysfunction in anxiety and anxiety disorders. Trends Neurosci (2012) 35(9):527–35. 10.1016/j.tins.2012.04.012 PMC3432139 22658924

[55] Gorka AX Torrisi S Shackman AJ Grillon C Ernst M Intrinsic functional connectivity of the central nucleus of the amygdala and bed nucleus of the stria terminalis. Neuroimage (2018) 168:392–402. 10.1016/j.neuroimage.2017.03.007 28392491 PMC5630489

[56] Sporns O Contributions and challenges for network models in cognitive neuroscience. Nat Neurosci (2014) 17(5):652. 10.1038/nn.3690 24686784

